# Evaluation of the Osteoconductive Capacity of Micro-Hydroxyapatite and Nano-Hydroxyapatite Associated with Beta-Tricalcium Phosphate: Experimental Study in Critical Calvarial Defects in Rats

**DOI:** 10.3390/jfb17100534

**Published:** 2026-10-21

**Authors:** Marcos Eduardo Gomes Alves, Igor Da Silva Brum, Izabella Sol, Maria Eduarda Asman Gonçalves, João Paulo Schmitt Lopes, Carlos Nelson Elias, Lucio Frigo, Ana Lúcia Rosa Nascimento, Jorge José de Carvalho, Daniela Ponzoni

**Affiliations:** 1 Department of Biomaterials and Oral Biology, School of Dentistry, University of São Paulo, São Paulo 05508-000, Brazil; marcos.gomes@unesp.br; 2 Faculty of Dentistry, State University of Rio de Janeiro, Rio de Janeiro 20551-030, Brazil; 3 Department of Diagnosis and Surgery, Araçatuba School of Dentistry, São Paulo State University, São Paulo 16015-050, Brazil; izabella.sol@unesp.br (I.S.); daniela.ponzoni@unesp.br (D.P.); 4 Materials Science Department, Instituto Militar de Engenharia, Rio de Janeiro 22290-270, Brazil; elias@ime.eb.br; 5 Basic Sciences Department, Faculdade de Odontologia da APCD, São Paulo 02011-000, Brazil; luciofrigo@uol.com.br; 6 Laboratory of Stem Cell Research, Histology and Embryology Department, Biology Institute, State University of Rio de Janeiro, Rio de Janeiro 20550-170, Brazil

**Keywords:** nano-HA/β-TCP, biomaterials, bone regeneration, calcium phosphates

## Abstract

Among the biomaterials used in grafting techniques for bone rehabilitation and subsequent dental implant placement, biphasic ceramics have emerged as promising alternatives, prompting investigations into their structural and biological properties. The objective of this study was to evaluate bone repair in critical-size calvarial defects in rats using two particulate biphasic ceramics composed of micro- or nano-hydroxyapatite associated with βtricalcium phosphate in different proportions. Seventy-two Wistar rats were used, distributed into four groups: blood clot (BC); GenPhos^®^ 70/30 (GP), containing micro-HA; Blue Bone^®^ 80/20 (BB), containing nano-HA; and Bio-Oss^®^ (BO). In each rat, a critical defect was created in the calvaria, filled according to the group and covered with a collagen membrane. The animals were euthanized at 7, 14, and 28 days. Histomorphometric analysis was performed, quantifying newly formed bone, biomaterial, and connective tissue, in addition to microcomputed tomography (Micro-CT), adopting *p* < 0.05, and qualitative histological analysis. In the Micro-CT analysis, no significant differences in bone volume were observed among the groups, although the BB group exhibited a more pronounced trabecular microarchitecture. Histomorphometrically, all biomaterial groups were superior to BC (*p* < 0.001). The BB group showed similar mean new bone formation (45.03%) compared to the BO group (46.62%), at 28 days, with no statistically significant difference, exhibiting an intraparticular osteoconduction pattern. The GP group demonstrated greater resorption dynamics (17.22%), whereas BO remained the most stable (44.22%). The BC group showed the highest connective tissue persistence (68.07%). It is concluded that the biphasic ceramics and the xenogeneic biomaterial promoted defect closure, with nano-hydroxyapatite (BB) demonstrating great qualitative performance in trabecular organization and initial vascularization, indicating that the nanometric scale and the bioactivity of β-TCP favor a dynamic tissue replacement in critical defects.

## 1. Introduction

The incorporation of biomaterials for guided bone regeneration (GBR) represents a significant advance in implant dentistry [1]. In this context, research has intensified to develop bone substitutes that exhibit ideal properties for bone neoformation associated with osteopromotion using membrane barriers, as conceptualized in the GBR process for atrophic jaws [2].

Fundamentally, there are two types of materials used in this process: grafts and bone substitutes. Bone graft is a living tissue capable of promoting the consolidation of new bone [3]. Bone substitute, on the other hand, is a synthetic or natural material, often composed of a mineralized matrix without viable cells, which aims to achieve clinical results similar to those of the graft [4]. As for classification, bone substitutes are divided into three main groups: allogeneic, xenogeneic, and synthetic (alloplastic), while the graft is strictly classified as autogenous [5]. One of the most researched materials is bovine xenograft, due to its satisfactory osteoconductive capacity and long-term volume maintenance [6]. However, the literature warns that prion disease, or Creutzfeldt–Jakob disease (CJD), has a highly infectious bovine and human transmissibility and can lead to motor dysfunction, dementia, and death [7]. Although there are no reports of patients being exposed to prion protein via bovine xenograft material, biological plausibility cannot be ruled out, given the protein’s resistance to sterilization and the consequent risk of contamination [7]. In this scenario, the focus of research has shifted to synthetic materials composed of polymers, ceramics, or metals [8,9]. These do not require a donor area, pose no immunogenic risks, and do not contain pathogens. The ceramics used in bone remodeling are bioactive and composed of calcium and phosphate ions, which serve as precursors for chemical bonds in the formation of regenerative compounds [10]. Calcium phosphate (CaP) bases have a composition similar to that of the inorganic phase of bone, making this composite more suitable than other options, such as sulfonated bases [11]. In addition, CaP allows for different chemical arrangements and ionic structures, exemplified by hydroxyapatite (HA) (Ca_10_(PO_4_)_6_(OH)_2_) and beta-tricalcium phosphate (βTCP) (Ca_3_(PO_4_)_2_)_11_. By combining HA and β-TCP, obtained via sintering at high temperatures or chemical processes such as calcium deficiency in acidic solution [12], a biphasic ceramic (BCP) is formulated. It is possible to control physicochemical properties during the sintering of CaP-based materials, such as HA particle size and the HA-to-β-TCP ratio [13]. Consequently, factors such as biocompatibility, porosity, solubility, and osteoconductivity are affected by variations in BCPs. In other words, the physical properties of the substitute determine distinct biological activities during regeneration: materials with a higher HA concentration exhibit slower reabsorption due to their bioinertia, whereas higher concentrations of β-TCP result in ionic dissolution and faster reabsorption by the organism [13,14,15]. Furthermore, changes in HA particle size influence bioactive behavior. Reducing the scale from micrometric to nanometric (nano-HA, 1 to 100 nm) increases surface area, favoring contact with blood and facilitating cellular activities such as angiogenesis and protein adsorption [16,17]. This accelerates remodeling and provides a trabecular microarchitecture favorable to load compression [18], thereby overcoming one of the current limitations of synthetics: the lack of adequate mechanical resistance to functional loads [18]. Given the above, this study aims to examine the correlation between the osteoconductive activity of different commercial bone substitutes and their CaP and HA particle sizes. The aim is to provide clinicians with in vivo fundamentals, through the creation of critical defects—surgical wounds that do not heal on their own—in the calvaria of Wistar rats, to provide statistical and qualitative data for choosing the synthetic material that best resembles the desired outcome during bone neoformation in the routine clinical practice of professionals.

The objective of the present study is to evaluate the osteoconductive capacity of two particulate biphasic ceramics with varying concentrations of hydroxyapatite and beta-tricalcium phosphate, in relation to the micrometric and nanometric sizes of hydroxyapatite particles, in critical calvarial defects in Wistar rats.

## 2. Materials and Methods

This experimental study was conducted in accordance with the ethical principles for animal experimentation, with approval from the Ethics Committee on Animal Use (CEUA) of the São Paulo State University “Júlio de Mesquita Filho”, Faculty of Dentistry of Araçatuba (FOA—UNESP), according to the ARRIVE Guidelines, under protocol No. 251-2024, protocol date 22 May 2024.

The sample size calculation was performed using the website http://estatistica.bauru.usp.br/calculoamostral/ta_comparacao_multipla_independentes.php (accessed on 16 February 2024), following the previous study by de Puttini et al. [19], considering the comparison between more than 2 Means with Independent Groups (ANOVA) and the primary outcome: quantitative analysis of the percentage of new bone formation in relation to the groups and experimental period of 7 days.

Based on the primary outcome, the minimum difference to be found between the control group (Blood Clot) and the experimental group (Biphasic Ceramic) was 2.79. Regarding the standard deviation estimate in the experimental group (biphasic ceramic), the value obtained was 1.36. Therefore, considering an alpha error rate of 5%, a beta error rate of 20%, and a power of 80%, the sample size was 6 animals per analysis time across the 4 proposed groups, for a total of 72 animals.

### 2.1. Biomaterials Used

The biphasic ceramic GenPhos^®^ XP (Baumer, Mogi Mirim, SP, Brazil) is composed of microHA and β-TCP (obtained in ovens at 800 °C) in a 70:30% ratio, CaP ratio 1.7. They are non-cytotoxic, have an estimated reabsorption time of 7–9 months with the smallest granules measuring 0.5–0.75 mm, have a porosity of 60–70%, are hydrophilic (can be mixed in physiological saline and with blood), and are sterilized by gamma radiation [20].

The biphasic ceramic Blue Bone^®^ (Regener biomaterials, Curitiba, PR, Brazil) is composed of nanoHA and β-TCP (obtained in ovens at 800 °C) in an 80:20% ratio, CaP ratio 1.7. They are not cytotoxic, have an estimated resorption time of 9 months with the smallest granules measuring 0.25 to 1 mm, have 70–80% porosity, are hydrophilic (can be mixed in physiological saline and blood), and are sterilized by gamma radiation [21].

The xenogeneic biomaterial Bio-Oss^®^ (Geistlich Pharma, Wolhusen, Switzerland) is composed of lyophilized bovine bone, with an expected resorption and bone neoformation time between 4–6 months. The smallest particles, measuring 0.25–1 mm, were used in the research. It has osteoconductive action. Considered as a positive control in the study in question due to the literature reports with results similar to autogenous graft11.

The implanted biomaterials were covered with a resorbable acellular biological membrane (Green Membrane^®^, Regener biomaterials, Curitiba, Brazil) [22,23] to ensure adequate physical barrier formation during particle retention until clot stabilization and to prevent interference from soft tissue cells in critical defects, ensuring that the same condition was created in all experimental groups. This includes both the negative control group, which contains only the clot, and the groups using biphasic ceramics and xenograft. The choice of collagen membrane (Green Membrane^®^) was based on recognized studies in scientific literature that characterize the biological activity of this material, as reported by Brum et al. [23]. This membrane is composed of type I collagen and is not cross-linked, Table 1.

### 2.2. Experimental Design

**Figure 1:**
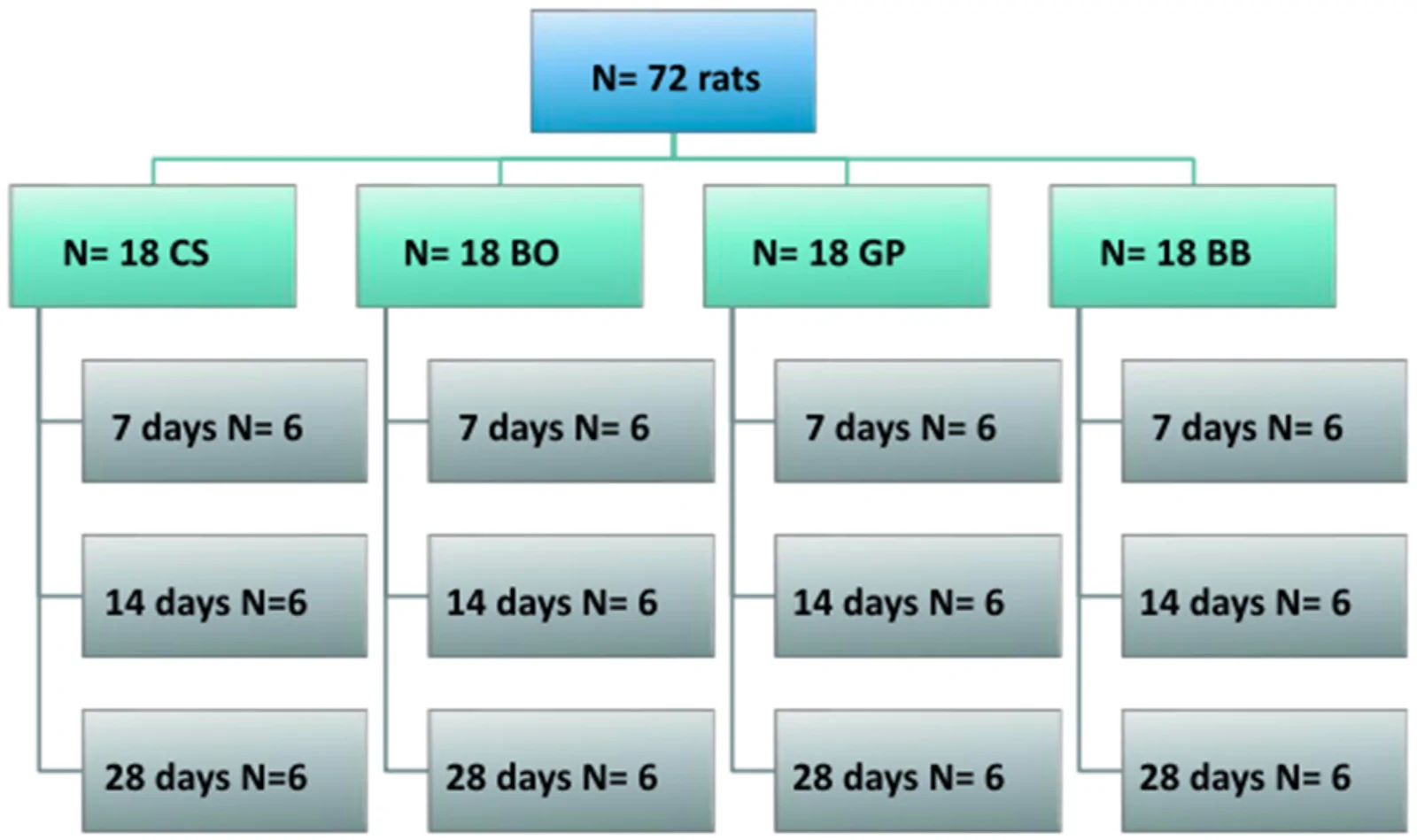
Flowchart of the experimental design, including all animals (N = 72), showing the randomization and allocation of animals into four experimental groups (N = 18 per group): biphasic ceramic groups (BB and GP), Bio-Oss® group (BO), and blood clot group (CS). Each group was subdivided according to the three experimental time points (7, 14, and 28 days), with N = 6 animals per group at each time point. The different colors are used for visual organization only and do not represent additional experimental variables.

A total of seventy-two (72) male Wistar rats (*Rattus norvegicus albinus*), aged 3 months and weighing between 450 and 500 g, were utilized. The animals were obtained from the Central Animal Facility of the Araçatuba School of Dentistry (UNESP) and housed in the Department of Diagnosis and Surgery at the same institution under controlled temperature and lighting conditions. Throughout the experiment, the rats received solid chow and water ad libitum. All subjects underwent the creation of a 5 mm critical-sized calvarial defect via osteotomy and were randomly assigned to one of four treatment groups:

- Control Group (CS): Defect filled with a blood clot, serving as the negative control group.
- Particulate Bovine Bone Group Bio-Oss^®^ (BO): Defect filled with granular bovine xenogeneic material Bio-Oss^®^ (Geistlich Pharma, Wolhusen, Switzerland), included for comparison among ceramics due to its volume maintenance during bone repair.
- Particulate Hydroxyapatite and β-tricalcium phosphate Group GenPhos^®^ XP (GP): Defect filled with granular synthetic hydroxyapatite/β-tricalcium phosphate (HA/β-TCP, 70/30) composite (GenPhos XP^®^, Baumer SA, Mogi Mirim, SP, Brazil).
- Particulate Hydroxyapatite and β-tricalcium phosphate Group Blue Bone^®^ (BB): Defect filled with granular synthetic hydroxyapatite/β-tricalcium phosphate (HA/β-TCP, 80/20) composite (Blue Bone^®^, Regener biomaterials, Curitiba, PR, Brazil). In all groups, the defects were covered with the Green Membrane^®^ cellular membrane (Regener Biomaterials, Curitiba, Brazil), Figure 1.

### 2.3. Surgical Procedure

**Figure 2:**
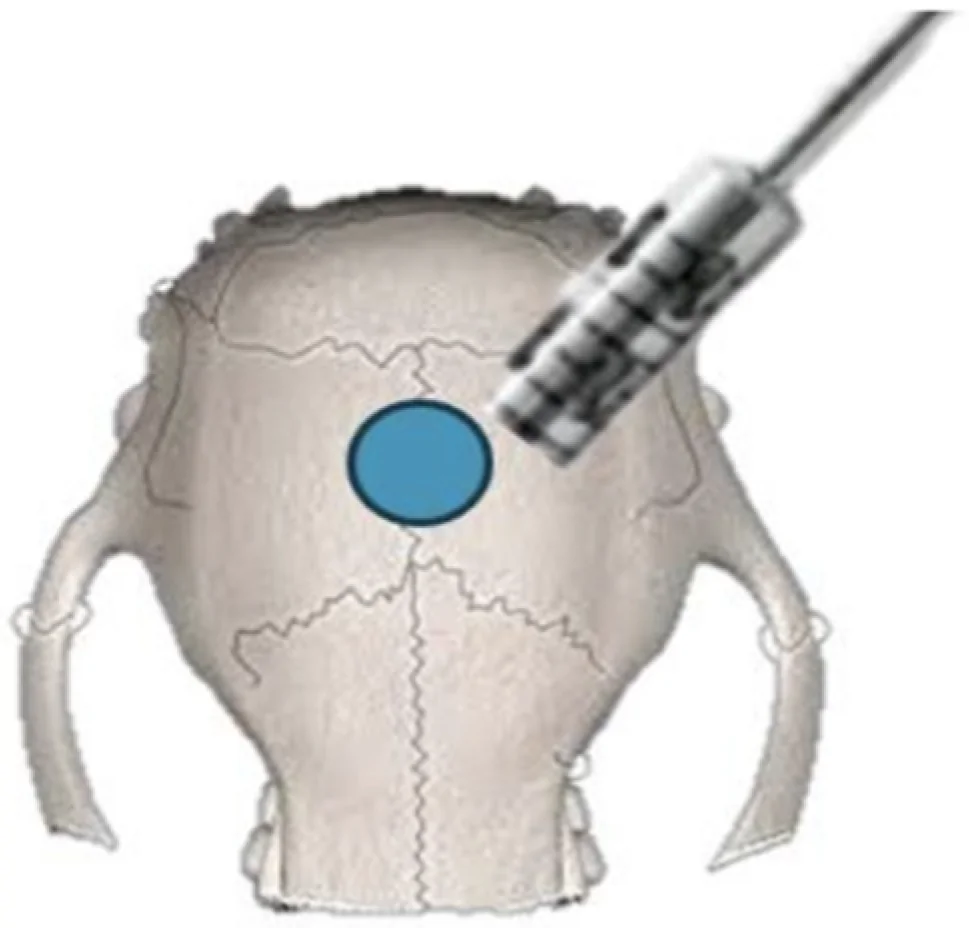
Representation of the critical-size defect site created in rat calvaria using a trephine bur (5 mm).

Animals were fasted for 12 h prior to the procedure. General anesthesia was induced following sedation by intramuscular administration of 80 mg/kg of 10% ketamine hydrochloride (Vetaset^®^—Fort Dodge Saúde Animal Ltda., Campinas, SP, Brazil) combined with 10 mg/kg of 2% xylazine hydrochloride (Dopaser^®^—Laboratório Calier do Brasil Ltd.a., São Paulo, SP, Brazil). Trichotomy of the fronto-parietal region was performed, followed by antisepsis with a topical polyvinylpyrrolidone-iodine solution (1% PVP-I; Riodeine Tópico, Rioquímica, São José do Rio Preto, SP, Brazil). Local anesthesia with 2% mepivacaine hydrochloride and 1:100,000 epinephrine (MEPIADRE^®^—DFL Indústria e Comércio S.A., Jacarepaguá, RJ, Brazil) was administered to supplement anesthesia and ensure hemostasis in the surgical field. Surgical access was achieved via a 2 cm linear V-shaped incision using a 15C scalpel blade (Feather Industries Ltd.a., Tokyo, Japan) mounted on a No. 3 handle (Hu-Friedy^®^, Chicago, IL, USA), followed by mucoperiosteal flap elevation and exposure of the parietal bone. A bicortical osteotomy of the central-middle region of the sagittal suture was performed using a trephine burr with a 5 mm internal diameter (Neodent^®^, Curitiba, PR, Brazil) attached to a 20:1 reduction contra-angle handpiece (Kavo^®^ do Brasil, Joinville, SC, Brazil) connected to a motor unit (BLM 600 plus, Driller^®^, Jaguaré, SP, Brazil), operating at 1200 rpm and a torque of 35 N. The cranial vault segment was carefully removed using a periosteal elevator to preserve the integrity of the dura mater. The critical-sized defect was then filled according to the four proposed treatment groups (CS, BO, GP, BB) and covered with an absorbable membrane (Green Membrane^®^, Regener Biomaterials, Curitiba, PR, Brazil), Figure 2.

**Figure 3:**
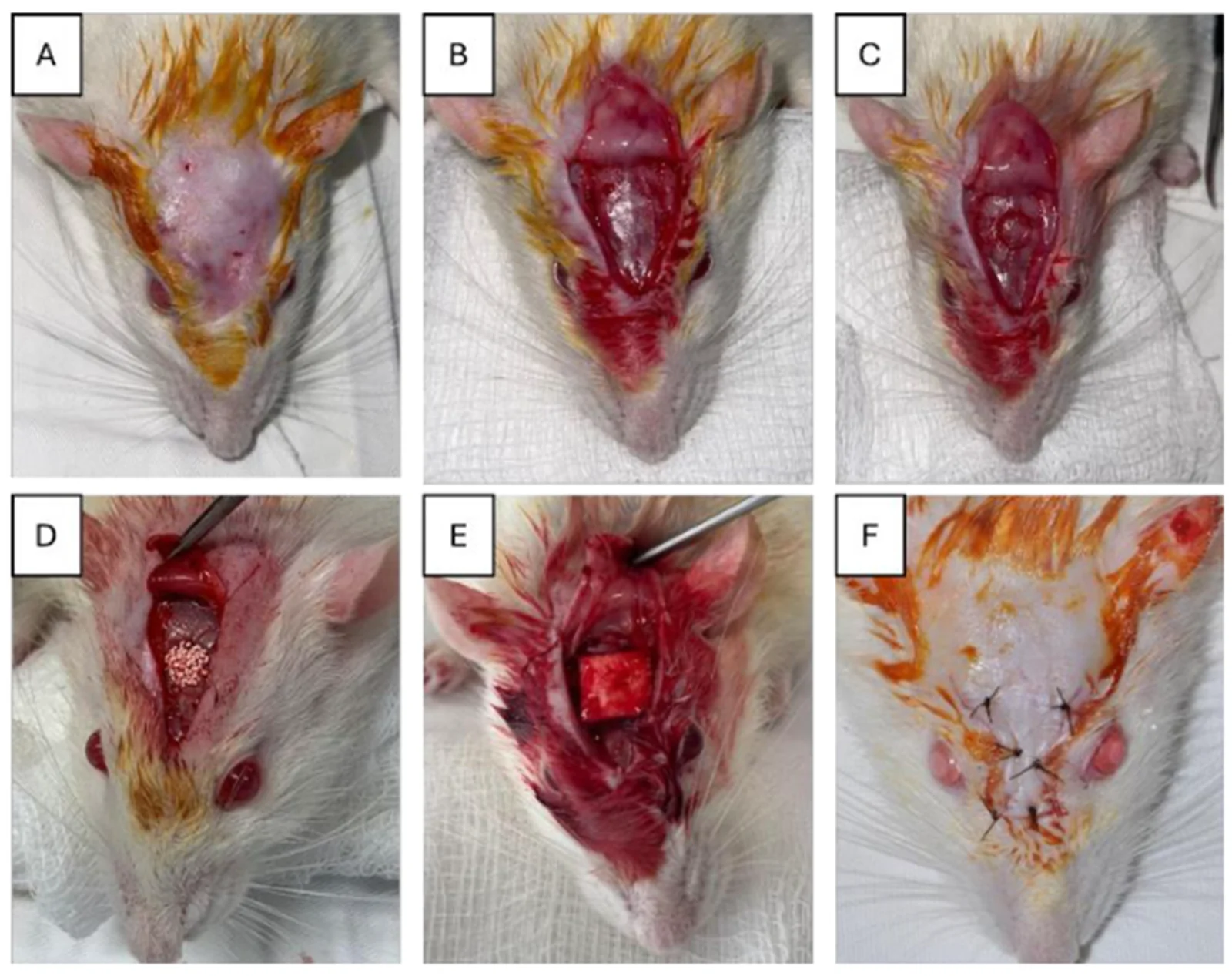
(**A**) Shaving + antisepsis; (**B**) incision + flap elevation; (**C**) critical-size defect; (**D**) filling the defect with biomaterial; (**E**) membrane placement; (**F**) suture.

In the immediate postoperative period, animals received intramuscular injections of 0.1 mL/kg Pentabiótico (Fort Dodge Saúde Animal Ltd., São Paulo, Brazil) and 1 mg/kg dipyrone sodium (Ariston Indústrias Químicas e Farmacêuticas Ltd., São Paulo, Brazil), Figure 3.

**Figure 4:**
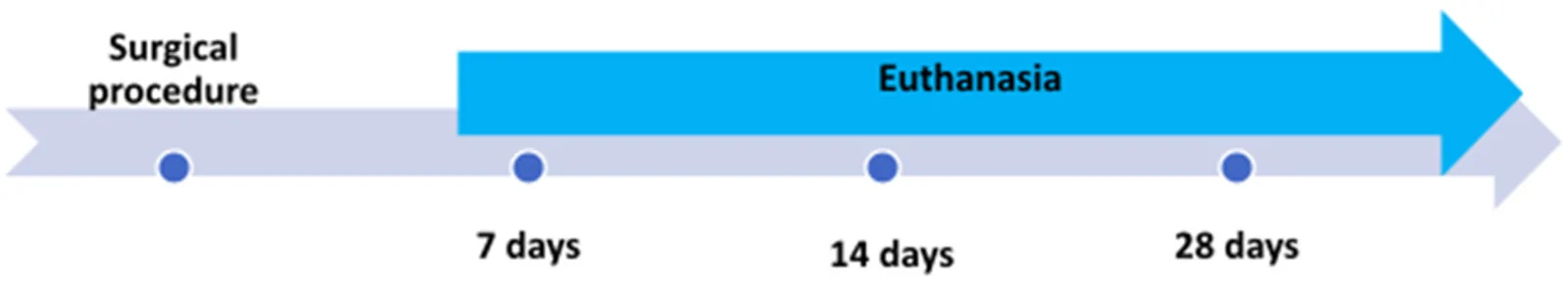
Flowchart of groups analysis time points: 7 days, 14 days, and 28 days.

At 7, 14, and 28 days post-operation, animals were euthanized via anesthetic overdose (three times the standard anesthetic dose) consisting of 10% ketamine (270 mg/kg; Vetaset^®^—Fort Dodge Saúde Animal Ltd.a., Campinas, Brazil) combined with 2% xylazine (30 mg/kg; Dopaser^®^—Laboratório Calier do Brasil Ltd.a., São Paulo, Brazil), administered intraperitoneally. Calvariae were then harvested and fixed in 10% neutral buffered formalin (Reagentes Analíticos^®^—Dinâmica Odonto-Hospitalar Ltd.a., Catanduva, Brazil) for 48 h. Specimens were rinsed in running water for 24 h and decalcified using 20% EDTA (ethylenediaminetetraacetic acid; Merck, Kenilworth, NJ, USA) dissolved in Milli-Q water at room temperature, with the solution changed weekly for approximately 9 weeks. Specimens were dehydrated using a graded series of alcohols (70%, 90%, 95%, and 100%), with the solution changed every hour, then cleared in xylene and embedded in paraffin to obtain 5 µm sections for hematoxylin and eosin (H&E; Merck & Co., Inc., Kenilworth, NJ, USA) staining. Two samples from each group were set aside, fixed in formalin for 24 h, and stored in 70% alcohol for micro-computed tomography (micro-CT) analysis. These samples were subsequently decalcified to facilitate further analysis, Figure 4.

### 2.4. Assessment of the Inflammatory Profile

The entire inflammatory cell lineage, with particular attention to lymphocytes and blood vessels, was quantified by selecting three slides from each animal at 7 and 14 days. Three sections per slide were photographed using a light microscope (DM 4000 B; Leica Microsystems, Wetzlar, Germany), a color image processor Leica QWin V3 image analysis software (Leica Microsystems Imaging Solutions., Cambridge, UK), a color camera DFC 500 digital camera (Leica Microsystems, Heerbrugg, Switzerland), and a computer (Intel Core i5, Intel Corp., Santa Clara, CA, USA; Windows 10, Microsoft Corp., Redmond, WA, USA). Images were captured at the center of the defect, as well as to the right and left of the center. At 100× magnification, a grid containing 130 (13 × 10) points was applied using the “grid” tool in the ImageJ (version 1.54g; National Institutes of Health, Bethesda, MD, USA), and every inflammatory cell or vessel cluster intersecting the grid points was counted [24].

### 2.5. Histological and Histomorphometric Analysis

**Figure 5:**
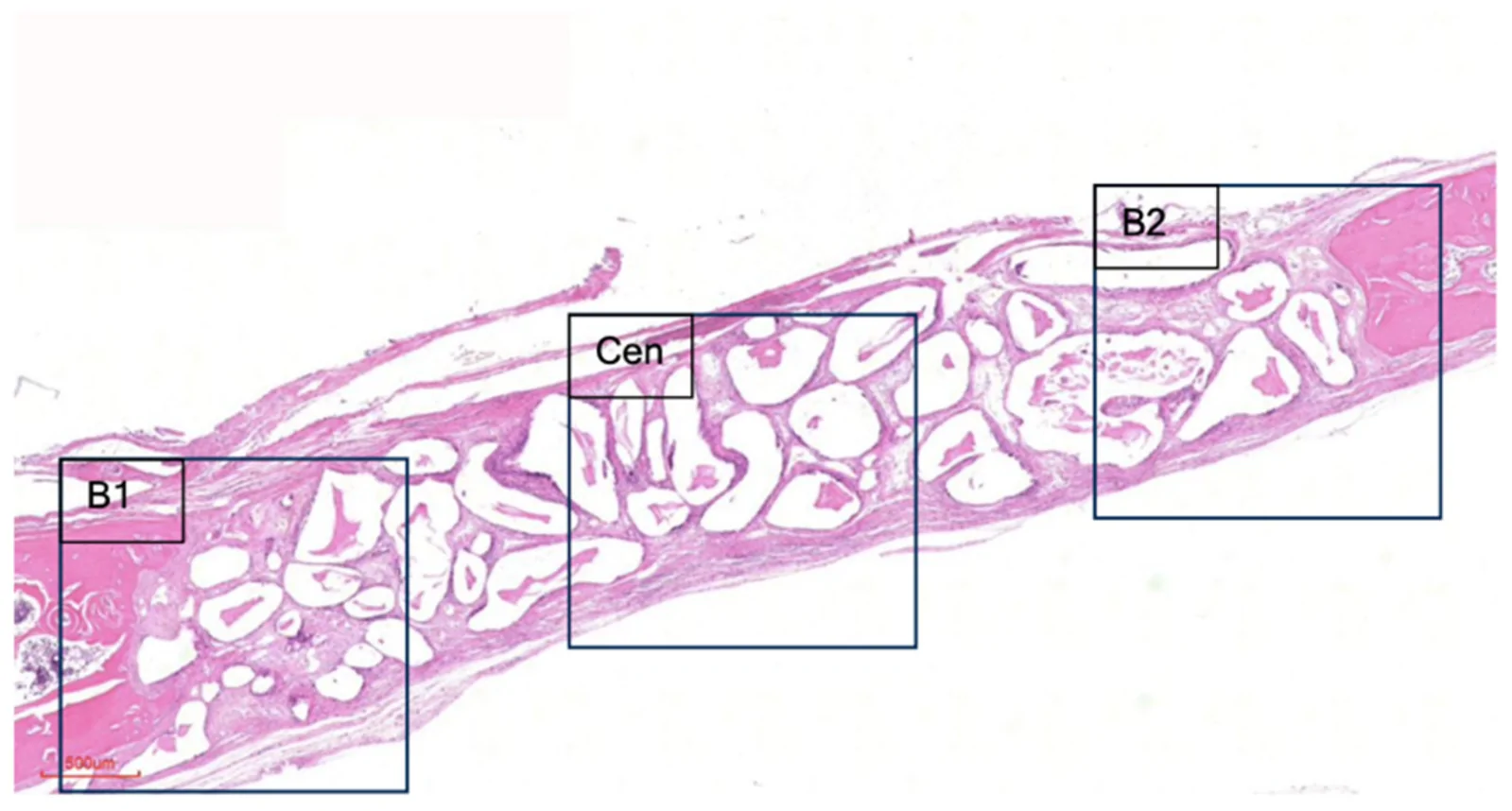
Division of the region of interest (ROI) for each group by time point: (B1) Edge 1; (Cen) Center; (B2) Edge 2.

A single trained examiner, blinded to group allocation, conducted the qualitative histological and histomorphometric analyses. For qualitative evaluation, three histological sections were prepared from each sample in a lateral-to-lateral orientation, covering the wound edge regions (B1 and B2) and the center (Cen), and imaged using digital scanning (MoticEasyScan One, Motic, Hong Kong, China) at 14× magnification [25] (Figure 5).

**Figure 6:**
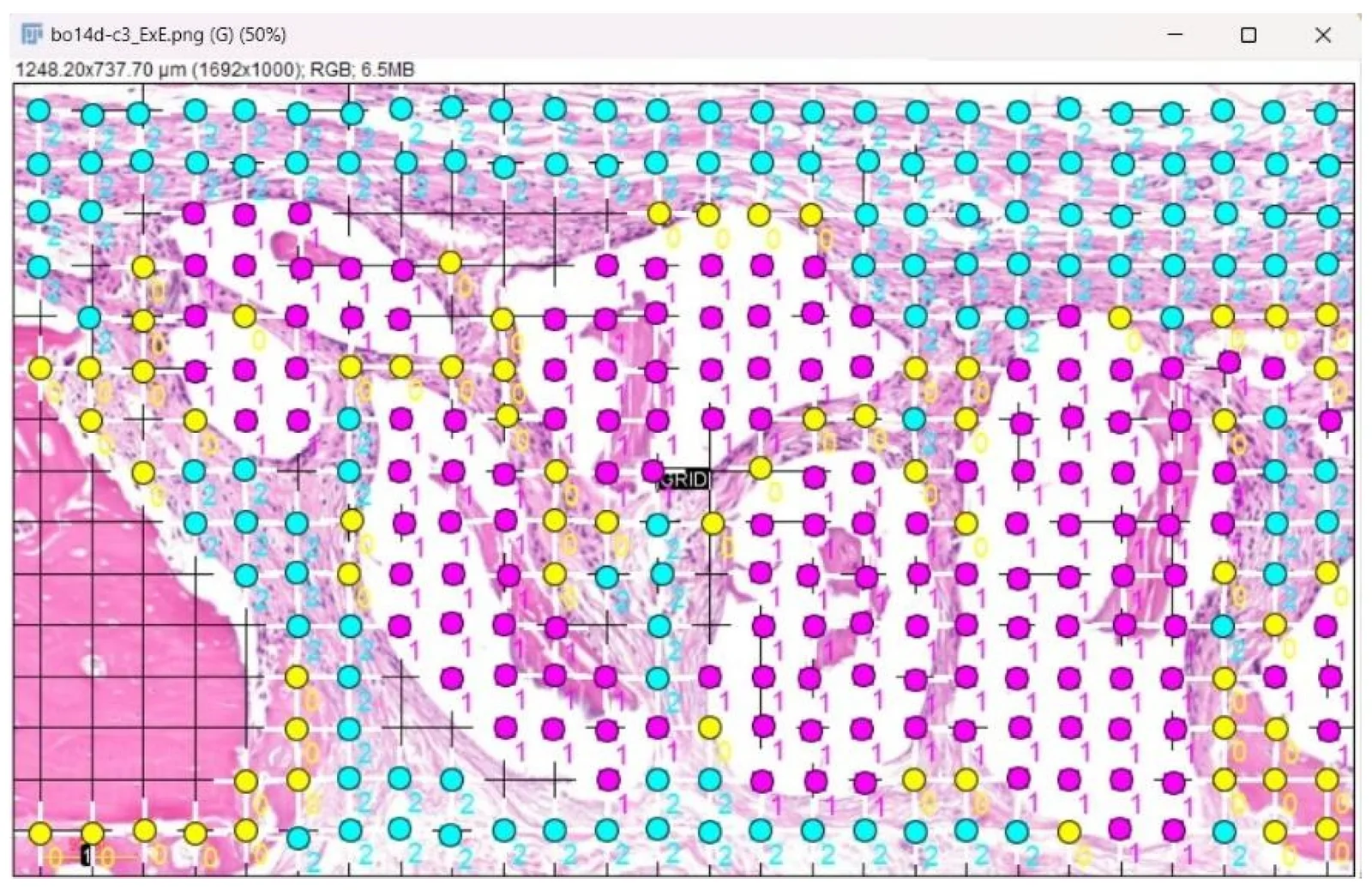
Counting stage of the points (391) in the thirds of each group (yellow for newly formed bone, pink for residual biomaterial, and blue for connective tissue).

Quantitative histomorphometric analysis was conducted using ImageJ^®^ software (version 1.54g; U.S. National Institutes of Health, Bethesda, MD, USA). A grid comprising 391 intersection points (17 by 23) was superimposed on images of each region (ExE, Cen, and ExD), Figure 5. This technique followed stereological principles, with each point classified according to the underlying structure using the following color-coding scheme: yellow for newly formed bone, pink for residual biomaterial, and blue for connective tissue, Figure 6 [26].

Point-counting data were organized in Microsoft Excel spreadsheets, and the area of each component was expressed as a percentage using the formula: (Number of points for the structure/Total number of points for the defect) × 100. The data were then exported to GraphPad Prism 8.0 software, Figure 6.

### 2.6. Microtomographic Analysis (Μct)

For microtomographic (µCT) evaluation, two specimens from each euthanasia time-point group were randomly selected. Specimens were fixed in 10% formaldehyde for 48 h, washed in running water for 24 h, and stored in 70% alcohol prior to evaluation using a Skyscan 1174 micro-computed tomography scanner (Bruker, Kontich, Belgium). The scanning parameters included a pixel size of 11.87 µm, 50 kVp, 0.5 mm aluminum filter, 0.6° rotation step, and 180° rotation arc. Acquired images were imported into NRecon Reconstruction Software (version 1.6.10.2, Bruker microCT, Kontich, Belgium) for three-dimensional (3D) grayscale reconstruction of the calvariae. Red lines delineated the area to be reconstructed, and a green line indicated the center of the region of interest. Following 3D image acquisition, DataViewer software (version 1.5.1.2 64-bit, Bruker microCT, Kontich, Belgium) was used to determine the volume of interest (VOI) by aligning the specimen in the sagittal, coronal, and axial planes and calculating the average volume to define the region of interest (ROI, 5 × 5 mm^2^). The slices were saved for bone analysis using CTAnalyzer software (CTAn, version 1.15.4.0+, 64-bit, Bruker microCT, Kontich, Belgium). The observed volume was assessed by binarizing the sample, yielding the total volume and the volumes of the biomaterial and newly formed bone phases. The morphometric parameters evaluated included bone volume (BV), bone volume fraction (BV/TV), trabecular thickness (Tb.Th), trabecular number (Tb.N), trabecular separation (Tb.Sp), and total bone porosity (Po.tot). All data were tabulated for subsequent statistical analysis [25].

### 2.7. Statistical Analysis

Data were exported to GraphPad Prism 8.0 software (GraphPad Software, San Diego, CA, USA). The normality of the distribution was assessed using the Shapiro–Wilk test. Comparative analysis between groups (BO, BB, GP, and CS) and experimental time points (7, 14, and 28 days) was performed using two-way ANOVA. When the normality test indicated slight deviations, parametric analysis was retained because ANOVA is robust for biological samples with homogeneous variances. Multiple comparisons between means were conducted using Tukey’s post hoc test. For all analyses, statistical significance was set at 5% (*p* < 0.05).

## 3. Results

### 3.1. Inflammatory Profile Evaluation

**Figure 7:**
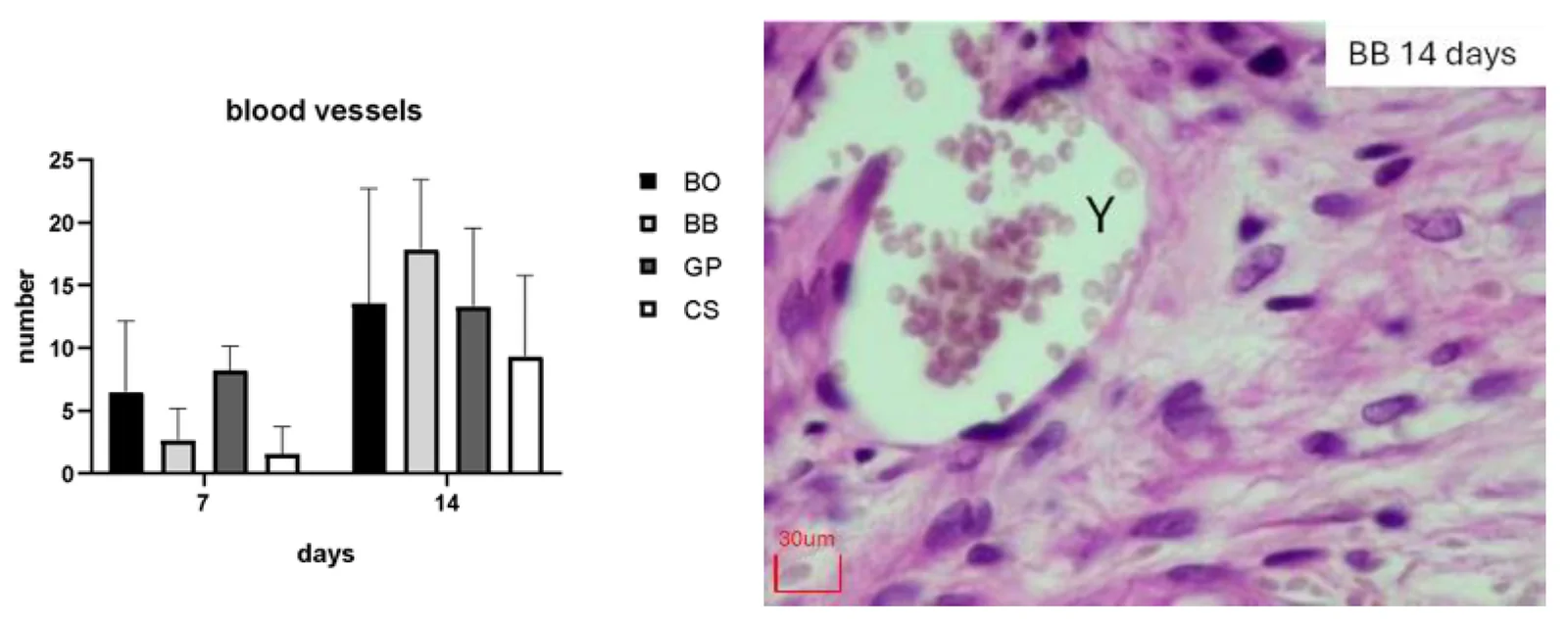
Blood vessel quantification and representative histology of BB at 14 days. On the (**left**), graph showing blood vessel count (mean ± standard deviation) at 7 and 14 days across BO, BB, GP, and CS groups, with an overall increase in vessel density at day 14 with the BB group achieving the highest mean count. On the (**right**), representative H&E-stained tissue section from the BB 14 days group complementing the quantitative findings, showing a well-formed blood vessel with erythrocytes inside the lumen (Y), lined by endothelial cells, and surrounded by connective tissue matrix with fibroblasts.

**Figure 8:**
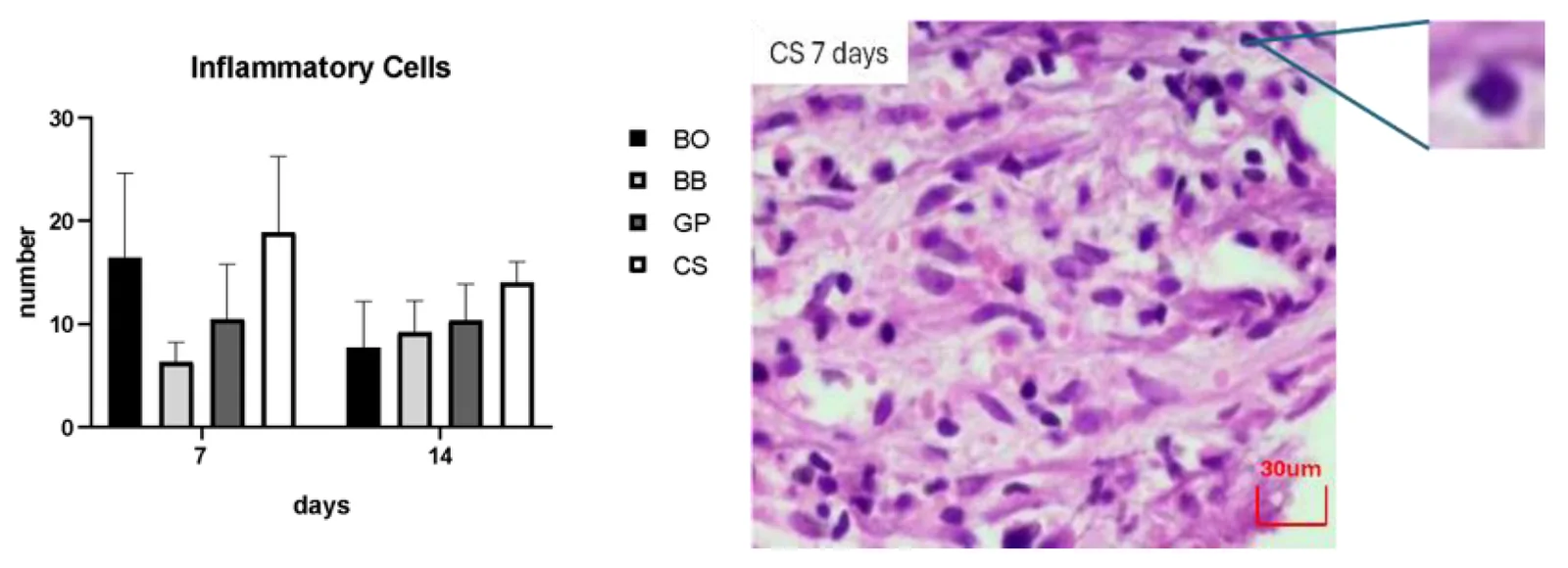
Inflammatory cell quantification and representative histology of the CS group at 7 days. On the (**left**), graph showing inflammatory cell count (mean ± standard deviation) at 7 and 14 days across BO, BB, GP, and CS groups, showing higher inflammatory cell density at day 7 in the CS group. On the (**right**), the representative H&E-stained tissue section from the CS 7 days group illustrates dense connective tissue infiltration by inflammatory leukocytes, with an inset highlighting a characteristic cell morphology.

Analysis of the inflammatory profile indicated that, across all experimental groups, the process progressed in a controlled manner consistent with the physiological phases of bone repair. No statistically significant differences were detected among the biomaterials in vessel count (*p* > 0.5998) or cellular infiltrate (*p* > 0.096), supporting the biocompatibility of all groups. At 7 days, a mild-to-moderate inflammatory infiltrate predominated, accompanied by early neoangiogenesis. The CS group exhibited the highest mean inflammatory cell count (18.88 ± 6.12), while the biomaterial-treated groups demonstrated a more organized cellular response around the particles: BO (14 ± 6.4), GP (11 ± 5.3), and BB (9 ± 3.7). In terms of vascularization, the BO group presented a mean of 12 ± 5.7 vessels, followed by GP (8 ± 3.2) and BB (2 ± 1.4). At 14 days, a transition to the proliferative phase occurred, with a progressive reduction in inflammatory infiltrate across all groups. The BB group demonstrated the greatest reduction in cell count (6 ± 2.5) and the largest increase in vessel count, reaching a mean of 17 ± 8.8 vessels. The GP (13 ± 5.2) vessels and BO (13 ± 5.3) vessels groups maintained a stable and mature vascular profile. During this period, inflammatory cellularity in the CS group also declined to 11 ± 4.7, indicating the natural resolution of the inflammatory process, Figure 7 and Figure 8.

### 3.2. Histological Evaluation

Across all experimental groups, the initial phase of new bone formation primarily originated from the residual bone stumps at the left and right margins of the surgical defect. The central region was predominantly composed of connective tissue, containing inflammatory cells and newly formed blood vessels.

In the control group (CS), disorganized connective tissue predominated, with minimal deposition of mineralized matrix between the bone edges (Figure 7). The biological membrane was present in all groups throughout the experimental periods and retained its structural integrity. Active angiogenesis was evident, as indicated by newly formed capillaries and blood cells, accompanied by a mild to moderate inflammation with lymphocytes dispersed throughout the white tissue stroma.

**Figure 9:**
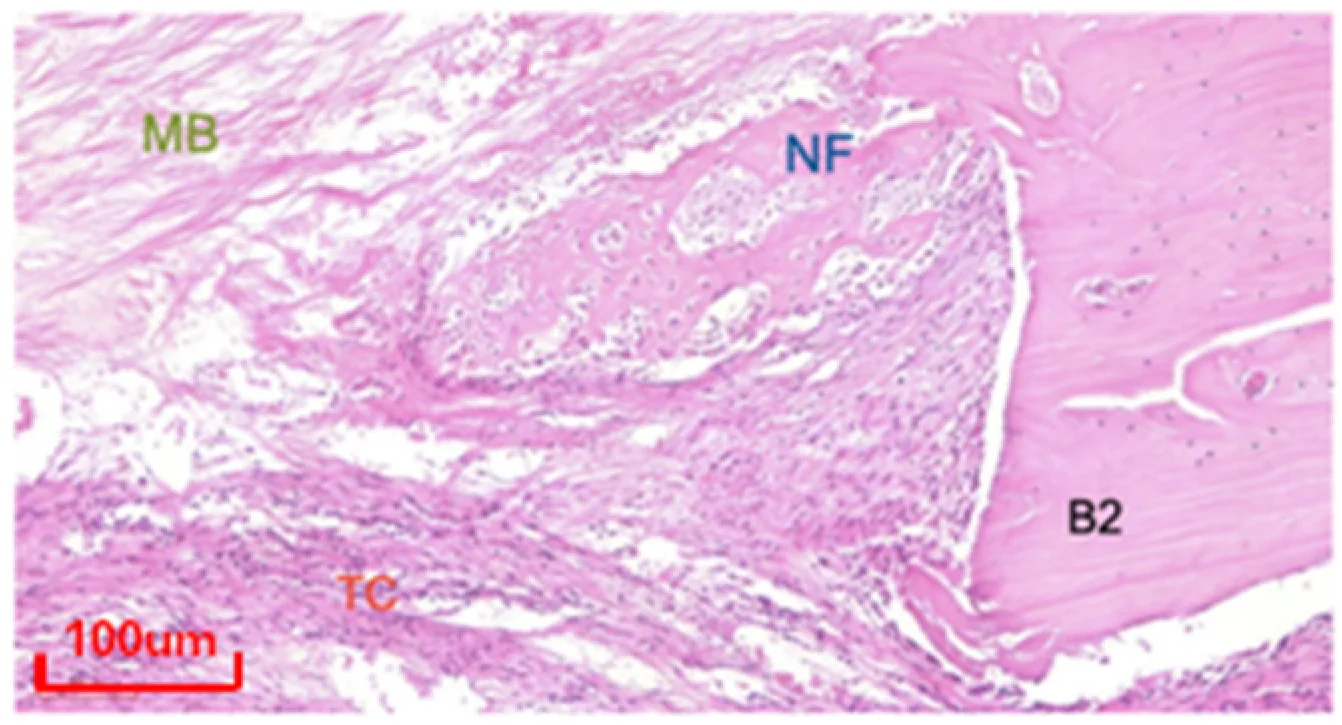
Histological section of the CS group at 28 days. Representative image showing new bone formation (NF) originating from the bone defect border (B2), located beneath the membrane (MB) and adjacent to the surrounding fibrous connective tissue (TC).

At 28 days, the CS group continued to exhibit a predominantly fibrous repair pattern. Although new bone formation increased slightly compared to the 7-day period, defect filling remained inadequate and displayed a pronounced concave morphology. At this time point, tissue organization was more apparent, with cells exhibiting circular morphology and rounded nuclei, consistent with pre-osteoblasts, in contrast to spindle-shaped fibroblasts embedded within a densely fibrous matrix, Figure 9.

In the Bio-Oss (BO) group, bovine hydroxyapatite particles were surrounded by a matrix undergoing mineralization, with osteoblasts positioned at the biomaterial–bone interface. Notably, colonization within the biomaterial was absent, indicating a pattern of new bone formation restricted to the particle periphery, as demonstrated in the central third of the histological assessment (Figure 8, 28 days, 14× magnification, 50 µm).

**Figure 10:**
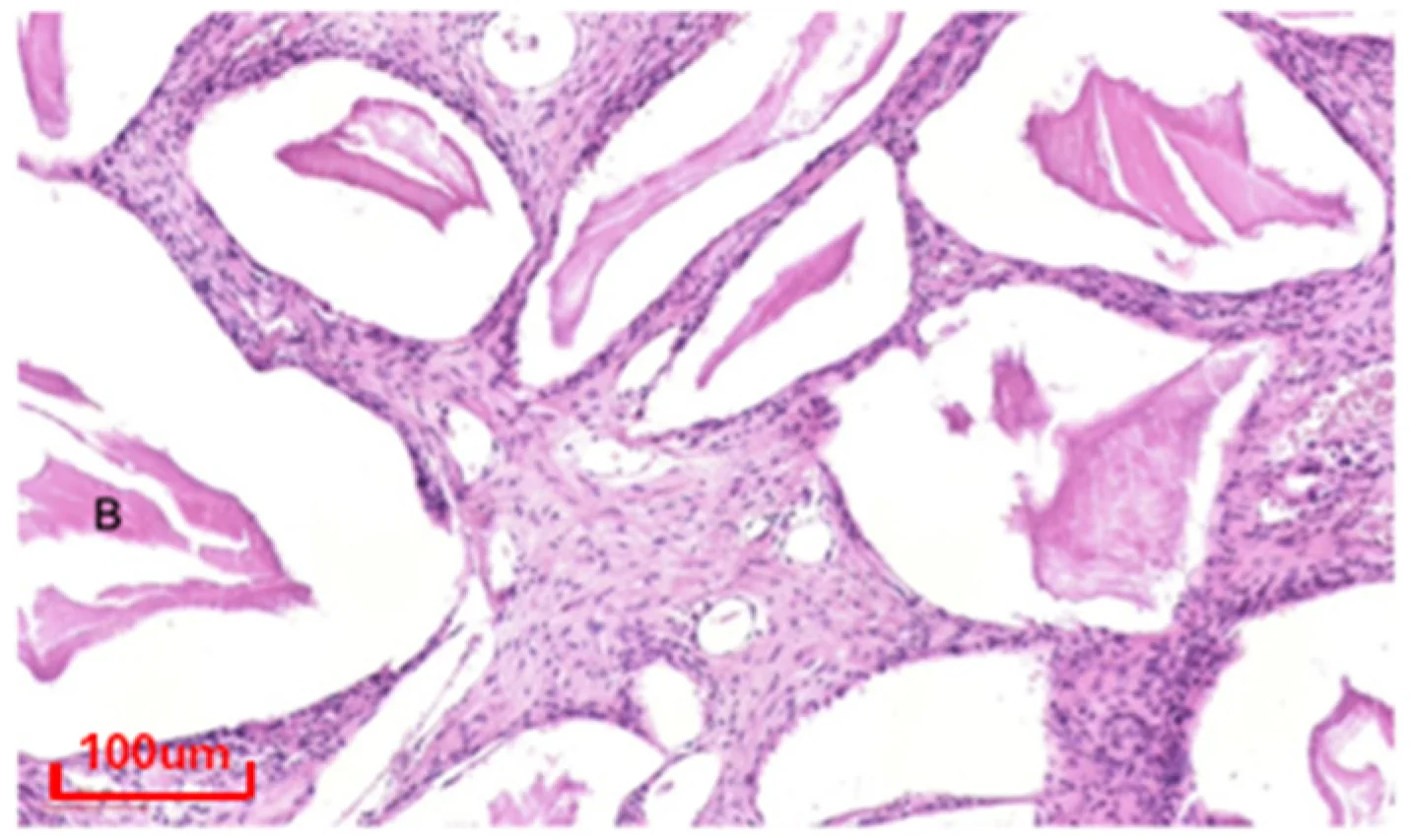
Histological section of biomaterial permanence in group BO at 28 days. The remaining biomaterial is surrounded by connective tissue and it is possible to see its lacuna with the biomaterial in the center (B).

At 14 days, all experimental groups exhibited centripetal progression of new bone formation from the surgical margins toward the critical zone of the defect. The BO group continued to display periparticular repair, with intact granules surrounded by well-organized bone trabeculae, although the defect remained unfilled. In contrast, the CS group showed a persistent deficit in lesion filling, marked by dense connective tissue and a concave architecture between defect margins, in contrast to the volumetric stability observed in the bovine xenograft group, Figure 10.

In groups treated with biphasic ceramics (GP and BB), a well-defined structural scaffold was consolidated, maintaining the defect’s volumetric architecture. In contrast to the bovine biomaterial, these groups demonstrated pronounced three-dimensional osteoconduction, as evidenced by progressive filling of inter-particle spaces and the presence of newly formed bone tissue islands within the lacunae of the HA particles, even at early time points.

**Figure 11:**
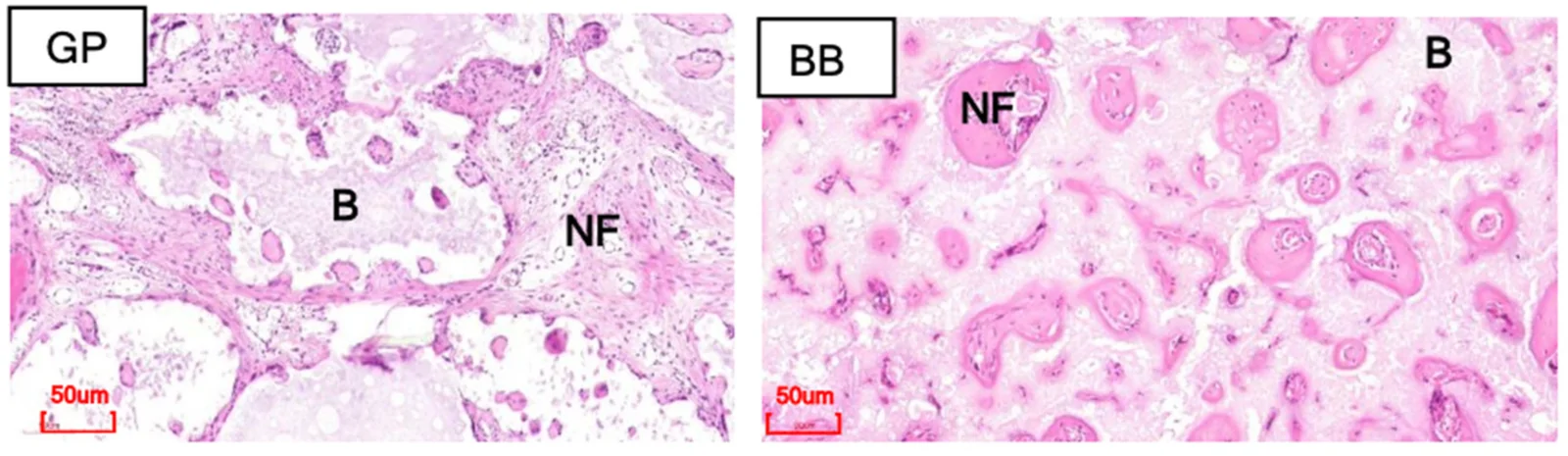
Histological sections of the defect center in GP and BB groups at 28 days. Representative images showing remaining biomaterial particles (B) and areas of new bone formation (NF). In the GP group, biomaterial (B) is present within lacunae bordered by newly formed bone matrix (NF). In the BB group, regions of new bone formation (NF) are visible alongside remaining biomaterial (B).

At 28 days, the regenerative process continued from the surgical margins, with growth fronts converging toward the central region in all groups. In these groups, the ceramic scaffold remained stable and was associated with extensive bone matrix deposition that enveloped and permeated the particles. Notably, the BB and GP groups exhibited a more widely distributed and organized trabecular pattern, particularly in the central thirds of the specimens, as shown in Figure 11 (28-day time point, 14× magnification/50 µm).

A progressive increase in the percentage of newly formed bone was observed across all experimental groups over time (*p* < 0.0001), reflecting the physiological progression of the repair process. At 7 days, mean values for newly formed bone remained low in all groups, consistent with the early phase of tissue repair. In the BO, BB, and GP groups, newly formed bone was primarily located adjacent to the biomaterial particles and bone stumps.

**Figure 12:**
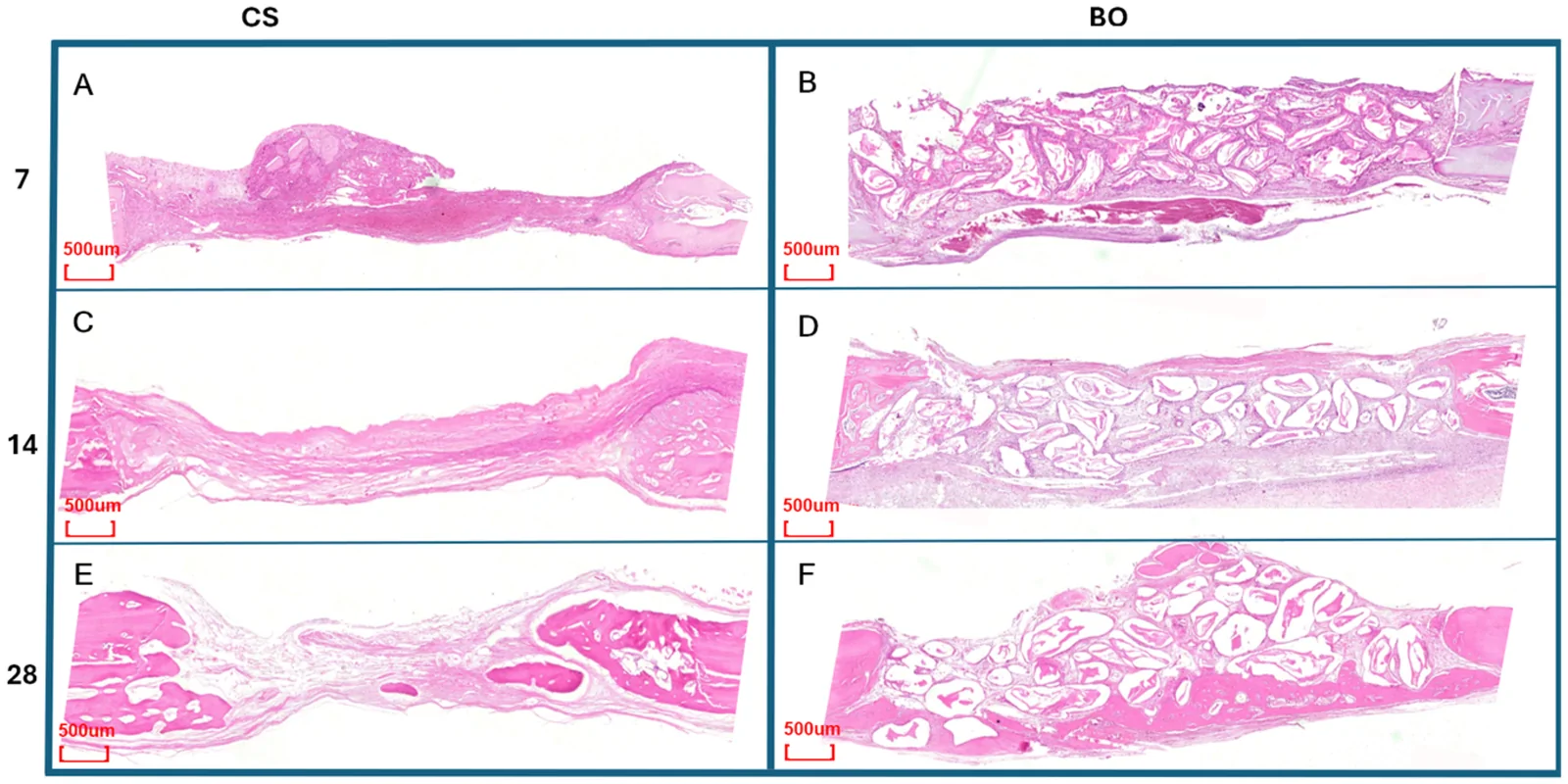

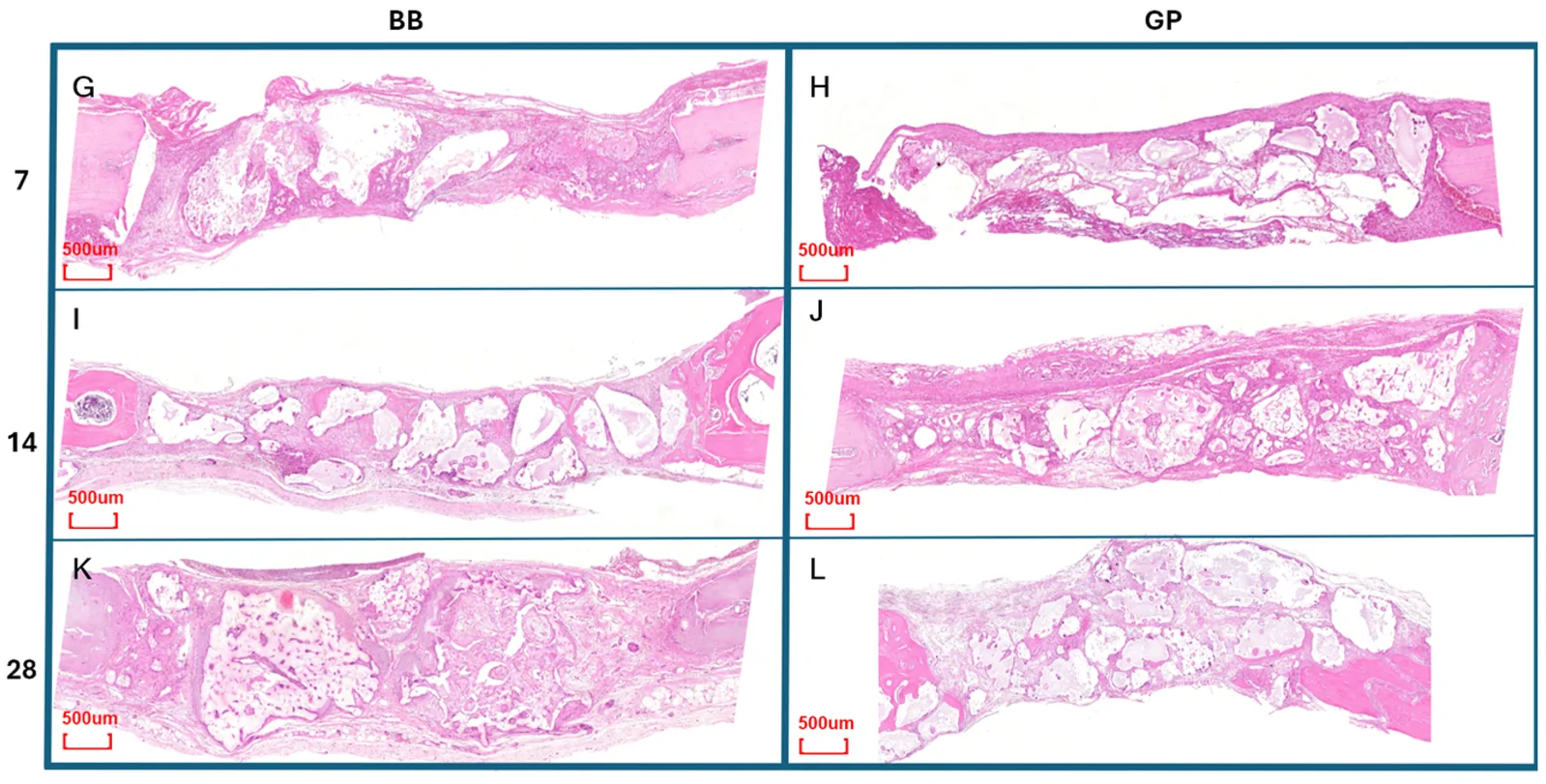
Histological aspects of all groups at 7, 14, and 28 time points, showing the maintenance of volume over time (**A**–**L**).

During this period, the BB group demonstrated the highest mean percentage of newly formed bone (12.5% ± 3), followed by the GP (11% ± 3) and BO (10.6% ± 3.4) groups, while the CS group exhibited the lowest value (7% ± 2). No statistically significant differences were detected between the biomaterial groups and the control group during this initial period (*p* > 0.05). At 14 days, mean values of newly formed bone increased in all groups, with bone formation advancing toward the center of the defect, Figure 12.

**Figure 13:**
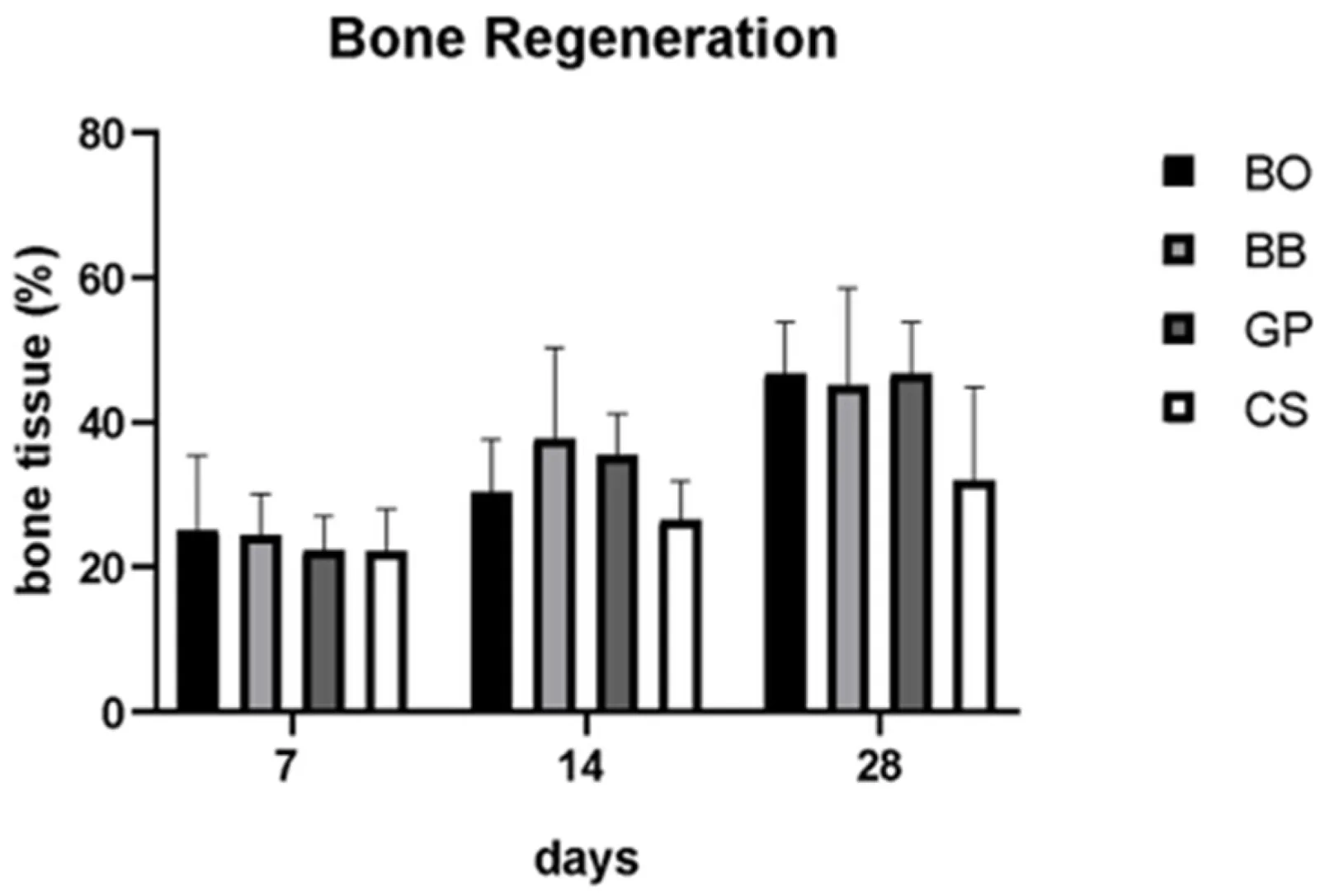
Assessment of new bone formation in experimental groups at the different analysis time points.

The BB (29% ± 4) and GP (27% ± 3) groups exhibited higher values compared to BO (23% ± 4) and CS (15.4% ± 3). Statistical analysis indicated no significant differences between treatments at this time point (*p* = 0.0514). At 28 days, peak bone formation was observed, with defects extensively filled by mineralized matrix, particularly in the biomaterial-treated groups. The BB (52.00% ± 5), GP (43% ± 4), and BO (46.6% ± 5) groups demonstrated the highest mean values, whereas the CS group showed the lowest (32% ± 4) (Figure 13). Although numerical trends suggested greater bone formation in the groups with filler material, multiple comparison analysis indicated that the biomaterials performed equivalently to each other and to the control in terms of the final outcome (Table 2).

**Figure 14:**
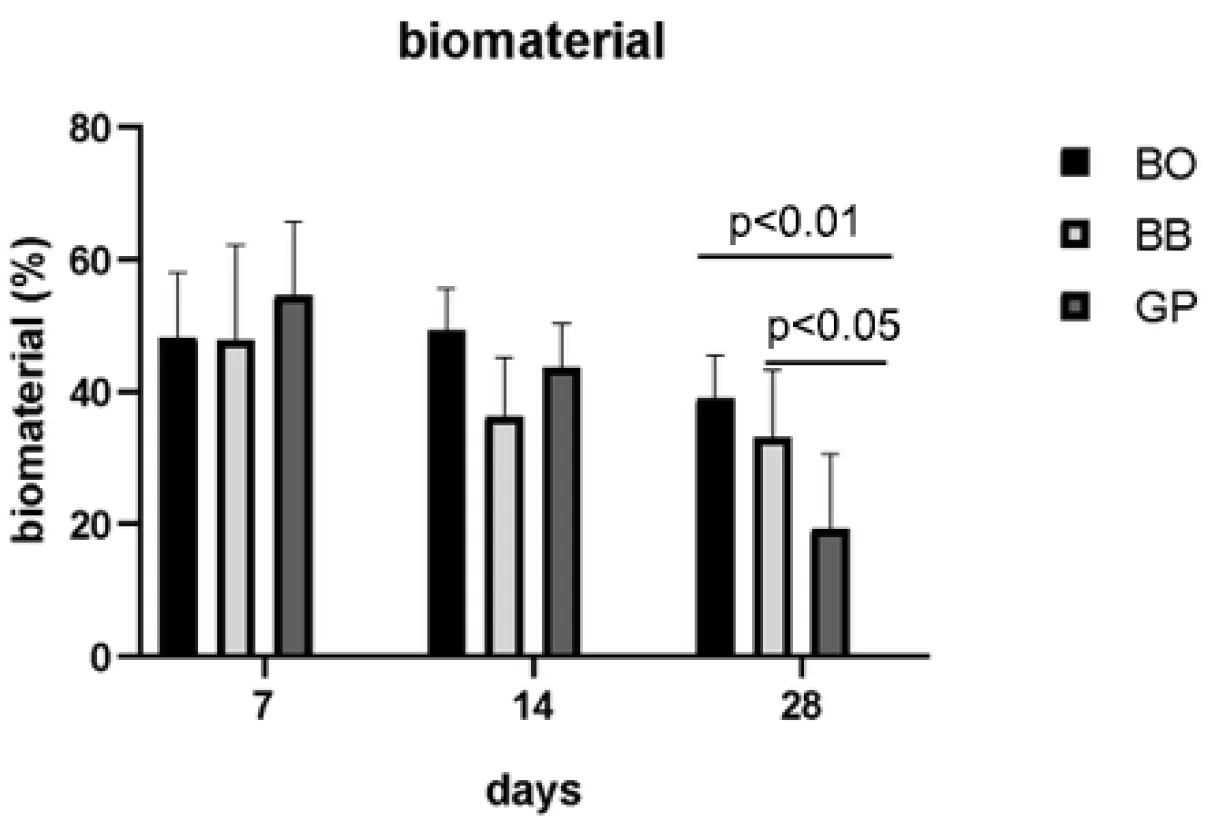
Assessment of the remaining biomaterial in the groups at the experimental time points.

Analysis of the percentage of remaining biomaterial demonstrated a strong dependence on experimental time in the BO, BB, and GP groups (*p* = 0.0003), reflecting the dynamics of resorption and gradual replacement by bone tissue. Statistically significant differences were also observed among the types of biomaterials used (*p* < 0.0001). At 7 days, all treated groups exhibited a high presence of biomaterial, with the GP group showing a mean of 54.5% ± 5, the BB group 48% ± 5, and the BO group 46% ± 3. During this initial period, no statistically significant differences were detected among the materials (*p* > 0.05), consistent with the graft stabilization phase at the recipient site. At 14 days, a reduction in the volume of remaining material was observed, with the GP group at 35.5% ± 4 and the BB group at 41% ± 5. The BO group maintained more stable values (45.5% ± 5), indicating slower resorption compared to the biphasic ceramics. By 28 days, the reduction in biomaterial became more pronounced, and differences between the groups were evident. The GP group exhibited the lowest percentage of remaining material (17% ± 5), followed by the BB group (33% ± 4), while the BO xenogeneic graft maintained the highest retention rate within the defect (44% ± 5) (Table 3). Multiple comparison analysis confirmed that the GP group underwent significantly greater degradation than the BO (*p* < 0.01) and BB (*p* < 0.05) groups, highlighting the accelerated resorption dynamics of this specific ceramic. Figure 14.

**Figure 15:**
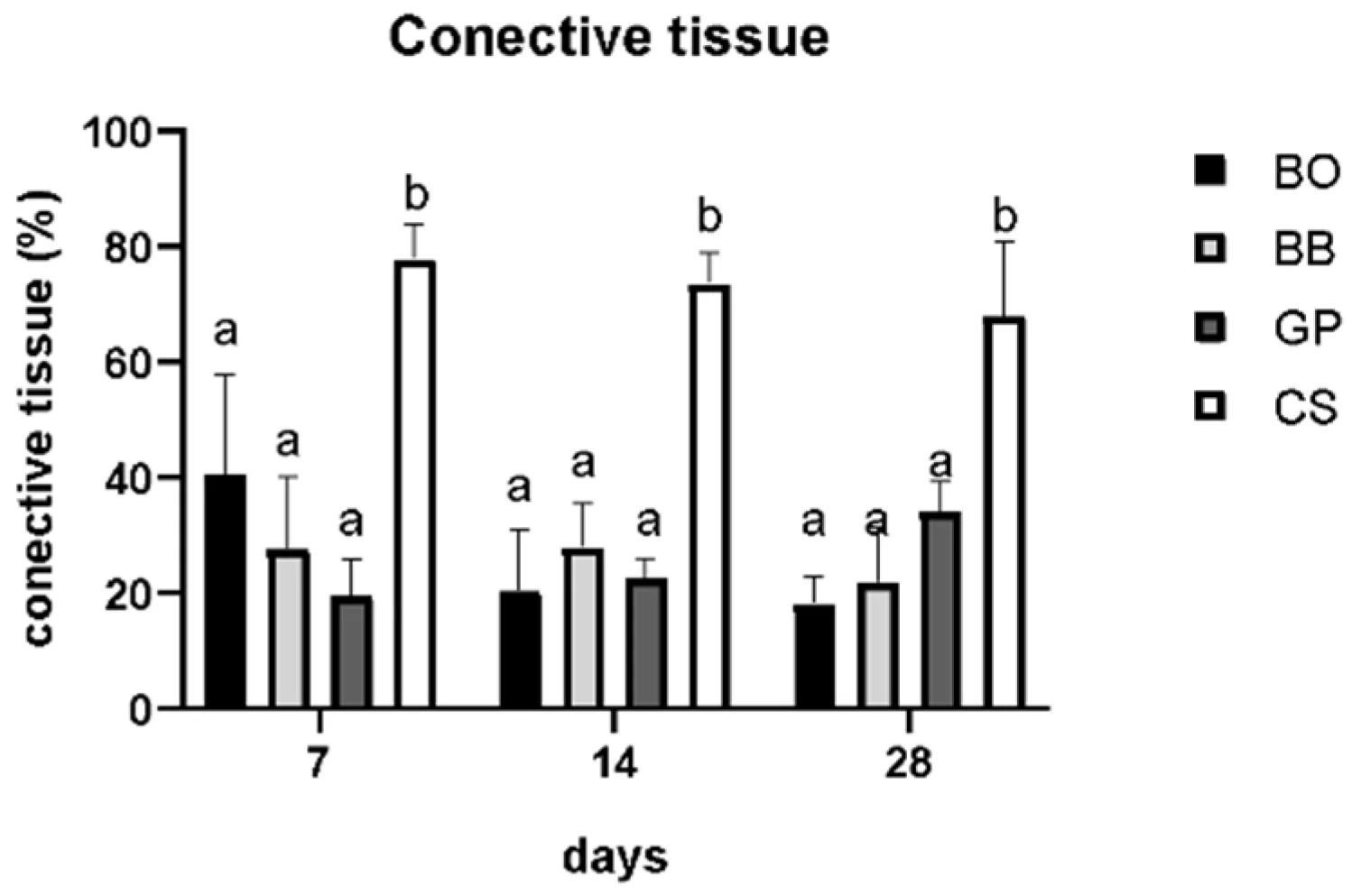
Evaluation of connective tissue in the groups at experimental time points. Different lowercase letters (a, b) indicate statistically significant differences between groups within the same experimental period (*p* < 0.001). Identical letters indicate no statistically significant difference.

Analysis of connective tissue percentage revealed an inverse relationship with new bone formation throughout the experimental period (*p* < 0.0001). A significant interaction between time and treatment was identified (*p* = 0.0012), indicating dynamic tissue replacement. At 7 days, connective tissue predominated in all groups, with the CS group exhibiting the highest mean (78% ± 6), which was statistically greater than the other groups (letter ‘b’ on the graph, *p* < 0.001). The biomaterial-treated groups—BO (40.5% ± 5), GP (32% ± 6), and BB (37.6% ± 5)—did not differ significantly from each other (letter ‘a’), indicating that these materials substantially reduced fibrovascular tissue compared to the initial phase. At 14 days, all groups showed a progressive reduction in the percentage of connective tissue: CS (67% ± 5), BO (29% ± 5), GP (35% ± 4), and BB (28% ± 4). The statistical difference between the control and biomaterial-treated groups persisted (‘b’ vs. ‘a’, *p* < 0.001), highlighting the continued presence of connective tissue in defects filled only by clot. By 28 days, connective tissue presence reached its lowest levels in the treated groups, remaining only between organizing bone trabeculae. The lowest values were observed in BB (20% ± 4), GP (18.6% ± 4), and BO (18% ± 4), with no significant differences among them (letter ‘a’). In contrast, the CS group maintained a high residual connective tissue percentage (68% ± 5), preserving statistical distinction (letter ‘b’, *p* < 0.001) (Table 4). These results confirm the limited capacity of clot alone to promote mineralized defect closure compared to the osteoconductive properties of biphasic ceramics and xenogeneic bone, Figure 15.

### 3.3. Micro-Computed Tomography Evaluation

**Figure 16:**
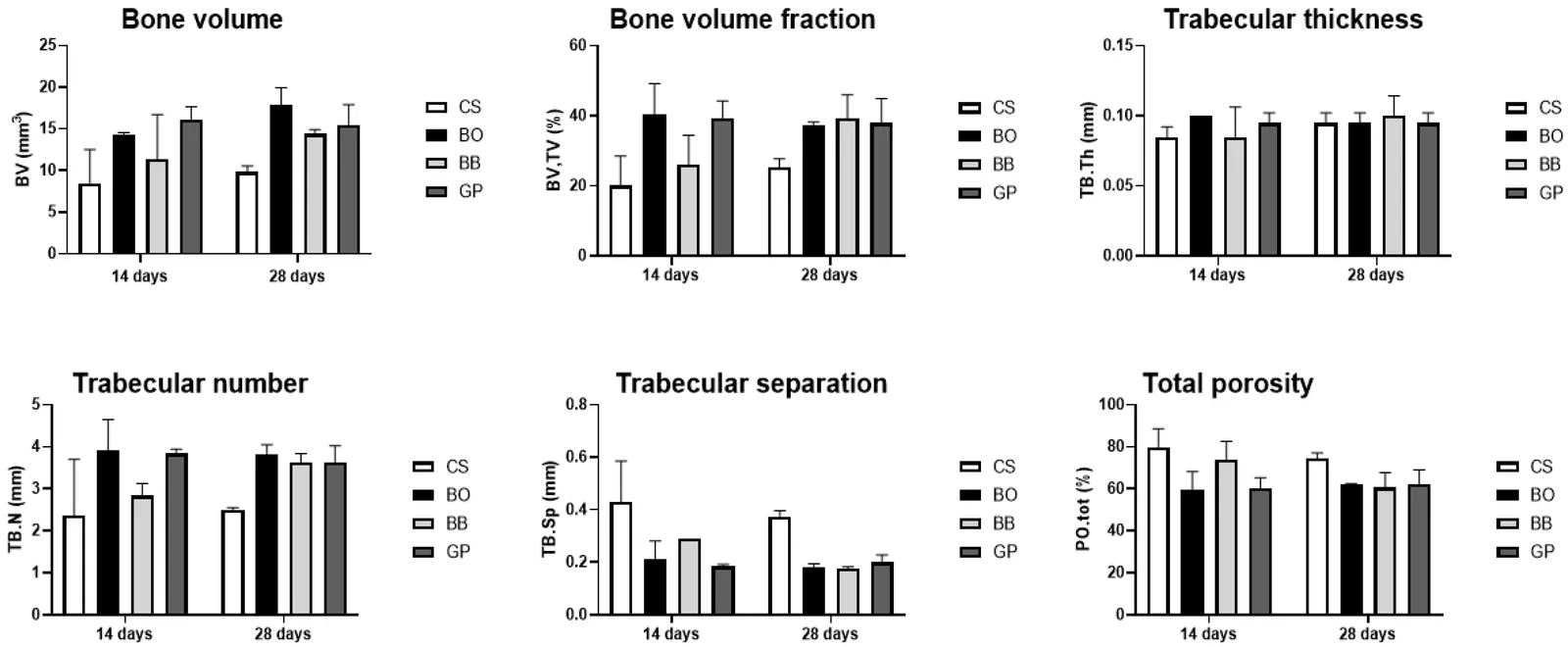
Analysis of morphometric parameters obtained by micro-computed tomography at 14 and 28 days.

Bone volume increased over time in all groups (Figure 16), with no statistically significant differences observed (*p* > 0.99). The BO group demonstrated the highest mean values at both time points (16.16 ± 4 mm^3^ and 17.91 ± 2 mm^3^), followed by the GP and BB groups (Table 5).

Regarding the Bone Volume/Total Volume (BV/TV) ratio, the BO and GP groups showed the highest BV/TV values at 14 days (40.55 ± 8% and 39.43 ± 4%, respectively). At 28 days, the BB group exhibited a significant increase (39.18 ± 6%), suggesting continued bone deposition (Table 6).

Regarding Trabecular Thickness (Tb.Th), the results showed that the groups remained stable across the time points, with mean values close to 0.10 mm and no statistically significant variation (Table 7).

Regarding the number of trabeculae (Tb.N), at 14 days, the BO and GP groups exhibited higher Tb.N than the other groups. At 28 days, the BB group showed a significant increase (3.64 ± 0.02), approaching the levels of the groups with the highest trabecular density (Table 8).

Regarding trabecular separation (Tb.Sp), a decrease in the spacing between trabeculae was observed in all groups over time, particularly in the CS group, which decreased from 0.43 ± 0.01 mm to 0.38 ± 0.02 mm. The GP group maintained the lowest values at both time points (Table 9).

Regarding the final micro-CT analysis, Total Porosity (Po.Tot) indicated that the CS group maintained the highest porosity at both time points (79.57 ± 9% and 74.51 ± 2%). In contrast, the other groups exhibited lower, relatively stable values. The GP group showed a slight increase at 28 days (from 60.46 ± 4% to 67.00 ± 0%) (Table 10).

## 4. Discussion

This study integrated microtomographic, qualitative histological, and histomorphometric data to demonstrate that all experimental groups exhibited progressive bone regeneration over the 28 days, albeit with distinct biological patterns among the evaluated biomaterials. Microtomographic analysis revealed a trend toward increased bone volume and gradual improvement in trabecular microarchitecture across all groups, with no statistically significant differences between them, indicating that, within this timeframe, bone remodeling was still in the early stage of structural maturation. These findings are fully consistent with the histological data, which showed that new bone formation began predominantly at the edges of the bone and progressed toward the center of the defect—a pattern typical of critical-sized calvarial defects [26,27]. Concurrently, histomorphometric analysis confirmed this behavior (albeit without statistical significance), showing a progressive increase in the percentage of newly formed bone over time across all groups, with higher values observed in the groups treated with biphasic ceramics (BB and GP), particularly at 28 days.

From a qualitative standpoint, the biphasic ceramics (BB and GP) suggest facilitated formation of a well-defined scaffold within the defect, with new bone developing both around and within the particles, a characteristic of three-dimensional osteoconduction [26,27,28,29]. This finding was quantitatively supported by the percentages of newly formed bone observed in these groups compared to BO and CS, especially at the final time point. In contrast, BO exhibited a distinct pattern, with bone formation occurring predominantly at the periphery of the particles and no intra-particle colonization—a behavior widely described in the literature for bovine hydroxyapatite xenografts [28,29]. The retention of higher percentages of residual biomaterial within the bone defect over time—particularly at 28 days—reinforces its slow-resorption characteristic, thereby promoting volumetric stability [27].

The biological differences observed between BB and GP, while subtle in the overall quantitative analysis, may be related, at least in part, to the nanoscale characteristics of hydroxyapatite. Previous studies have suggested that nanohydroxyapatite can more closely resemble the mineral phase of bone and may exhibit surface properties that favor protein adsorption and cell–material interactions. However, these mechanisms were not directly evaluated in the present study [30]. However, these mechanisms were not directly evaluated in the present study. The BB group, composed of nanohydroxyapatite and beta-TCP (80/20), exhibited superior microtomographic performance at 28 days, with higher relative bone volume (BV/TV) and denser trabecular microarchitecture. This phenomenon may occur because nanohydroxyapatite mimics the natural mineral crystals of human bone, which, according to Brum et al. [30], results in higher surface energy. This hydrophilic characteristic enhances the adsorption of key serum proteins that serve as anchorage points for osteoblasts. The presence of the nanoscale fraction promotes a larger contact area with proteins and cells, accelerates protein adsorption and osteoblastic differentiation, and intensifies local vascularization—factors considered determinants of bone repair quality, as highlighted by Brum et al. [23,30] and Feroz et al. [29]. This suggests the trend toward greater vascularization observed in the BB group at 14 days (17.88 ± 8). The intra-particle osteoconduction observed in the BB histology confirms that bone not only grows around the particles but utilizes them as an active biological bridge [31], transforming the defect into more organized and mechanically competent tissue (with reduced trabecular spacing) compared to bovine bone (BO), whose slow resorption and lack of nanoporosity limit this substitution dynamic [32].

Conversely, the GP group—composed of micro-hydroxyapatite combined with β-TCP (70/30)—while also demonstrating excellent osteoconductive performance, showed greater initial volumetric stability and slightly slower resorption dynamics compared to BB. Such behavior may be related to the larger size of the hydroxyapatite particles, which reduces the specific surface area and, consequently, the rate of ionic dissolution and tissue remodeling events [33].

The resorption dynamics observed in both the BB and GP groups are intrinsically linked to the biphasic nature of these compounds. The literature establishes that β-TCP exhibits significantly higher solubility than hydroxyapatite in a physiological environment [34]. Upon contact with biological fluids, β-TCP undergoes rapid ionic dissolution, releasing calcium (Ca^2+^) and phosphate (PO_4_^3−^) ions into the microenvironment of the bone defect [35]. This dissolution phenomenon increases scaffold porosity, facilitating angiogenesis and the migration of osteogenic cells. The formation of a biological apatite layer (carbonated hydroxyapatite) on the surface of the remaining HA particles is also part of the new bone formation process, enhancing local osteoconduction [36]. This phenomenon occurs with both xenogeneic and ceramic biomaterials. This process, known as dissolution-reprecipitation, allows the biomaterial surface to become chemically integrated with the newly formed bone, a phenomenon described as bioactivity [29]. In the BB group, the nanoscale HA enhances this transition by providing a higher surface energy, which serves as a nucleation site for the deposition of new calcium crystals, thereby mimicking physiological mineralization.

In contrast to BO, which acts primarily through passive osteoconduction due to its stable crystallinity and low turnover rate, biphasic ceramics operate via coordinated substitution. As β-TCP is resorbed, the newly mineralized bone utilizes the HA nuclei for structural organization. This dynamic nature explains why, despite no statistically significant difference in total bone quantity at 28 days, the microarchitectural quality and intra-particle integration in the BB and GP groups suggest long-term regenerative potential, characterized by a smoother transition between the biomaterial and the host tissue.

The BO group, in turn, maintained consistent performance across all time points, showing good bone volume and BV/TV values; however, its low resorption rate—evidenced by high percentages of residual biomaterial and the absence of intra-particle colonization—confirms that its primary biological function is to provide stable, bioinert structural support with slower remodeling [22,32].

Although the CS (Clot) group showed the lowest rates of new bone formation and the highest persistence of connective tissue (68.07% ± 5) at 28 days, the maintenance of a centripetal repair pattern and the preservation of the defect’s biological volume are attributable to the use of the Green Membrane^®^. According to the ultrastructural characterization by Brum et al. [30], this non-crosslinked type I bovine collagen membrane features a unique organization of five overlapping fiber layers, providing mechanical properties—such as ductility and strength—that are well-suited to its role as a barrier in guided bone regeneration procedures. Furthermore, the high wettability and surface roughness characteristic of this material promote rapid protein adsorption and fibroblast compatibility, allowing the membrane to act as an efficient selective barrier.

In the present study, the Green Membrane played a fundamental role in cell exclusion, preventing the invagination of overlying soft tissues and preserving the defect interior from the margins—albeit with a vertical reduction in space, as observed in Figure 11 at the 7-, 14-, and 28-day time points. However, quantitative data reinforce that these findings suggest that the presence of an osteoconductive scaffold may contribute to mineralized tissue formation within the defect, whereas the barrier membrane provides a protected environment for bone regeneration.

Connective tissue analysis revealed behavior inversely proportional to new bone formation, predominating in the early stages and progressively decreasing over time—particularly in groups treated with biomaterials—thereby demonstrating the physiological transition from granulation tissue to mineralized bone tissue. Furthermore, the angiogenesis observed across all groups, alongside the presence of erythrocytes, blood vessels, and lymphocytic infiltration, confirms an appropriate inflammatory response and the tissue viability of the experimental model.

Therefore, the integration of micro-CT, histological, and histomorphometric data demonstrates that biphasic ceramics—especially the nano-HA/β-TCP composite (BB)—exhibit biological behavior highly favorable to bone regeneration, characterized by dynamic tissue replacement, intense intra-defect osteoconduction, and progressive improvement in bone microarchitecture; meanwhile, BO maintains excellent stability but undergoes slower remodeling. These results reinforce the potential of biphasic ceramics as safe, effective, and bioactive alternatives for regenerative applications.

Despite the promising results obtained in this study, certain limitations must be considered when interpreting the findings. Firstly, the commercial biomaterials used in this study were not subjected to independent physicochemical characterization prior to their experimental application. Therefore, information regarding their phase composition, HAP/β-TCP ratio, granulation, and other physicochemical characteristics was based on the specifications provided by the manufacturers. Consequently, it was not possible to experimentally determine the individual contribution of each of these characteristics to the differences observed among the experimental groups. In particular, differences in composition and particle size were simultaneously present among the evaluated biomaterials, precluding the attribution of the observed effects exclusively to micro- or nanoscale particle dimensions. Future studies should therefore include a comprehensive characterization of the starting biomaterials, including their morphology, phase composition, specific surface area, and Ca/P ratio, to allow a more controlled comparison among the different materials.

The 28 day experimental period was selected based on established rodent bone repair models [24], as it corresponds to a critical phase of advanced matrix mineralization and tissue remodeling in small animals. Although extending the evaluation to 12 weeks would provide additional insights into late-stage bone maturation, 28 days served as an adequate and validated timeframe to capture the primary therapeutic outcomes and structural differences among the experimental groups. Additionally, the mechanical strength of the newly formed bone tissue was not assessed; this is a fundamental parameter for inferring its capacity to withstand functional loads and support the future placement of osseointegrated implants. It was also observed that individual variations in the histomorphometric values within each experimental group contributed to the lack of statistically significant differences between groups, despite consistent biological trends. In this regard, a larger sample size could have allowed better control of inter-individual variation, thereby increasing the statistical power of the analyses and enabling the detection of more subtle differences among the biomaterials. Furthermore, the micro-computed tomography analysis was conducted with a small sample size, which also limits the statistical power of comparisons regarding microstructural parameters. Thus, although the combined histological, histomorphometric, and micro-CT data indicate favorable biological behavior for the biphasic ceramics—particularly the nano-HA/β-TCP composite—future studies involving larger sample sizes, longer experimental periods, and biomechanical testing are required to confirm their clinical applicability, especially in situations requiring functional load-bearing support.

## 5. Conclusions

In conclusion, the particulate biphasic ceramics Blue Bone^®^ and GenPhos^®^, alongside the reference biomaterial Bio-Oss^®^, demonstrated high biocompatibility and effectiveness in promoting tissue repair in critical-sized bone defects. Although quantitative bone formation was statistically equivalent among all treated groups and the control at 28 days, the evaluated biomaterials played a critical structural role in maintaining defect volume and preventing soft-tissue collapse. Furthermore, the materials exhibited distinct physical–chemical properties and substitution dynamics throughout the healing process. Specifically, the GenPhos^®^ group presented more rapid resorption driven by the higher solubility of β-tricalcium phosphate (β-TCP).

On the other hand, the Blue Bone^®^ group demonstrated better qualitative tissue organization, favoring a more organized trabecular microarchitecture and a larger contact surface compared with the Bio-Oss^®^ and GenPhos^®^ groups.

Additionally, the Green Membrane^®^ effectively served as a functional physical barrier, preserving space and preventing soft-tissue invasion into the defect area.

Overall, these findings highlight that while bone volume recovery reaches a comparable endpoint across groups, the choice of biomaterial directly influences scaffold degradation kinetics, cellular response, and the structural quality of the newly formed tissue.

## Figures and Tables

**Table 1:** Comparison of the properties of the test biomaterials used in the groups. Source: Prepared by the author based on data provided by the manufacturers.

CHARACTERISTICS	GENPHOS^®^	BLUE BONE^®^
**OBTAINING**	MUFFLE FURNACE AT 800 °C	MUFFLE FURNACE AT 800 °C
**STERILIZATION**	GAMMA RADIATION	GAMMA RADIATION
**HA/β-TCP (%)**	70/30	80/20
**CYTOTOXICITY**	NON-CYTOTOXIC	NON-CYTOTOXIC
**HA PARTICLE SIZE**	MICRO-HA	NANO-HA
**GRANULATION**	0.50–0.75 mm; 0.5 cc; 0.6 g	0.25–1.0 mm; 0.5 cc; 0.25 g
**POROSITY**	60% to 70%	70% to 80%
**RESORPTION**	7–9 months	9 months
**HYDROPHILICITY**	YES	YES

**Table 2:** Mean and standard deviation of new bone formation (%).

Group	7 Days (%)	14 Days (%)	28 Days (%)
BO	10.6 ± 3	23 ± 4	46.6 ± 5
BB	12.5 ± 3	29 ± 4	52 ± 5
GP	11 ± 2	27 ± 3	43 ± 4
CS	7 ± 2	15.4 ± 3	32 ± 4

**Table 3:** Mean ± standard deviation of biomaterial presence (%) at 7, 14, and 28 days.

Group	7 Days (%)	14 Days (%)	28 Days (%)
BO	46 ± 3	45.5 ± 5	44 ± 5
BB	48 ± 5	41 ± 5	33 ± 4
GP	54.5 ± 5	35.5 ± 4	17 ± 5

**Table 4:** Mean ± standard deviation of connective tissue (%) at 7, 14, and 28 days.

Group	7 Days (%)	14 Days (%)	28 Days (%)
**CS**	78 ± 6	67 ± 5	68 ± 5
**BO**	40.5 ± 5	29 ± 5	18 ± 4
**BB**	37.6 ± 5	28 ± 4	20 ± 4
**GP**	32 ± 6	35 ± 4	18.6 ± 4

**Table 5:** Bone volume of the groups at 14 and 28 days.

BV				
mm^3^	CS	BO	BB	GP
14 days	8.48 ± 4	16.16 ± 4	11.33 ± 5	16.13 ± 1.5
28 days	9.82 ± 0.76	17.91 ± 2	14.41 ± 0.50	13.81 ± 0.00
*p*	>0.99	>0.99	>0.99	>0.99

**Table 6:** Bone volume fraction (BV/TV) of the groups at 14 and 28 days.

BV/TV				
%	CS	BO	BB	GP
14 days	20.1 ± 8	40.55 ± 8	25.97 ± 8	39.43 ± 4
28 days	25.31 ± 2	37.49 ± 0.69	39.18 ± 6	32.99 ± 0.00
*p*	>0.99	>0.99	>0.99	>0.99

**Table 7:** Trabecular thickness (Tb.Th) of the groups at 14 and 28 days.

Tb.Th				
mm	CS	BO	BB	GP
14 days	0.09 ± 0.01	0.10 ± 0	0.09 ± 0.02	0.1 ± 0.01
28 days	0.10 ± 0.01	0.10 ± 0.01	0.10 ± 0.01	0.09 ± 0
*p*	>0.99	>0.99	>0.99	>0.99

**Table 8:** Trabecular number (Tb.N) of the groups at 14 and 28 days.

Tb.N				
mm	CS	BO	BB	GP
14 days	2.37 ± 1	3.90 ± 0.07	2.83 ± 0.03	3.84 ± 0.1
28 days	2.49 ± 0.06	3.81 ± 0.03	3.64 ± 0.02	3.36 ± 0
*p*	>0.99	>0.99	>0.99	>0.99

**Table 9:** Trabecular separation (Tb.Sp) of the groups at 14 and 28 days.

Tb.Sp				
mm	CS	BO	BB	GP
14 days	0.43 ± 0.1	0.21 ± 0.07	0.29 ± 0.00	0.19 ± 0.01
28 days	0.38 ± 0.02	0.18 ± 0.01	0.18 ± 0.01	0.22 ± 0
*p*	>0.99	>0.99	>0.99	>0.99

**Table 10:** Total porosity (Po.Tot) of the groups at 14 and 28 days.

Po.Tot				
%	CS	BO	BB	GP
14 days	79.57 ± 9	59.44 ± 8	73.74 ± 8	60.46 ± 4
28 days	74.51 ± 2	62.18 ± 0.23	60.54 ± 7	67.00 ± 0
*p*	>0.99	>0.99	>0.99	>0.99

## Data Availability

The original contributions presented in the study are included in the article, further inquiries can be directed to the corresponding author.
